# Results of a unilateral lip lift for correction of a vertical disproportion in upper lip vascular anomalies

**DOI:** 10.4103/0970-0358.53006

**Published:** 2009

**Authors:** Shahriar Loghmani, Amir Momeni, Mohammad Eidy

**Affiliations:** Department of Plastic Surgery, Alzahra Hospital, Isfahan University of Medical Sciences, Isfahan, Iran

**Keywords:** Unilateral lip lift, Vascular anomalies, Macrocheilia

## Abstract

Congenital vascular anomalies most notably hemangiomas involving the lips, especially those which fail to regress, pose a difficult problem both for the surgeon and the patient. These lesions not only discolour the skin but may also distort the shape of the lip. When nonsurgical modalities fail to treat these lesions, surgery is chosen as the next viable step. In this article, we propose a well planned sequential surgical procedure for unilateral lesions. This approach in comparison with previously used procedures produces noticeably better results and fewer complications. During a period of 4 years, we treated 21 patients with vascular anomalies using the unilateral upper lip lift procedure to correct the vertical disproportion of commissures. Using this procedure, we managed to restore the normal form and symmetry of the upper lip in a majority of our patients with less obvious scarring and few complications.

## INTRODUCTION

Hemangiomas are considered to be the most common tumors in infants; they are three times more common in females than in males.[1–3] The hemangioma exhibits rapid proliferation and slow regression during childhood. They are a cause of great concern for parents; however, as most of them regress over time, the physicians should assure the parents of the benign nature of the lesion and the anticipated outcome of spontaneous involution.[4–7]

On the other hand, vascular malformations are comprised of abnormally formed vascular channels that are lined by quiescent endothelium. Although congenital, they are not always obvious at birth. They never involute and continue to grow even in adulthood.[5–8]

Vascular anomalies including hemangiomas and vascular malformations that involve the face and fail to regress have a detrimental psychological and functional effect on patients. Usually, these lesions not only cause a discoloration of the skin but they may also distort the normal shape of the face especially the form of the lips. Nonsurgical and surgical modalities are used for lesions that have failed to regress spontaneously.[8–12] In this article, we explain a new surgical technique that we refer to as a unilateral upper lip lift that is used for the treatment of vascular anomalies involving the unilateral upper lip.

## TECHNIQUE

One of the well established cosmetic surgical procedures for correction of a vertically long lip is a lip lift. We have developed a variation of this technique that we call the unilateral upper lip lift for correction of vertical disproportion in unilateral upper lip vascular anomalies. The principles of our technique are as follows.

With accurate measurements, the vertical height from the alar base to the commissure on both sides of the mouth angle is determined. After induction of local anesthesia (with injection of 2% Lidocaine with 1:100000 Epinephrine), a skin incision is made from the mid point of the base of columella and is extended into the perialar and nasolabial area; the incision in the nasolabial line is limited to 1 cm above the mouth angle [Figure 1a]. The incision is followed by dissection and debulking of the subcutaneous tissue up to the vermilion. The next step in our technique is an elliptical excision of the orbicularis oris muscle and suturing with proper tension using 4-0 polyglactin 910 (Vicryl^®^) or alternately we may only use plication of the muscle [Figure 1b]. Muscle plication or suturing helps to maintain long-term or permanent results. It is better to work on the muscle in the lower parts of the lip; in this area, the risk of denervation of the muscle is less than in the upper part of the lip.

**Figure 1a:**
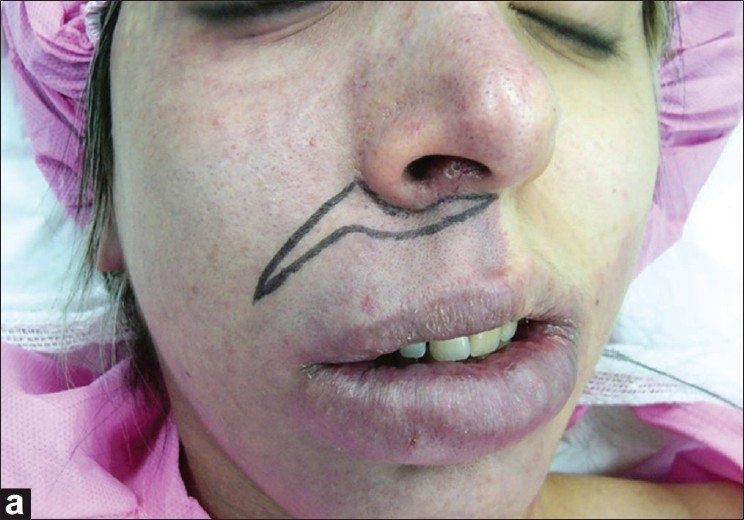
A unilateral lip lift skin incision is made from the subnasal area in midline and extended into the perialar and nasolabial area; the incision in the nasolabial line is limited to 1 cm above mouth angle

**Figure 1b:**
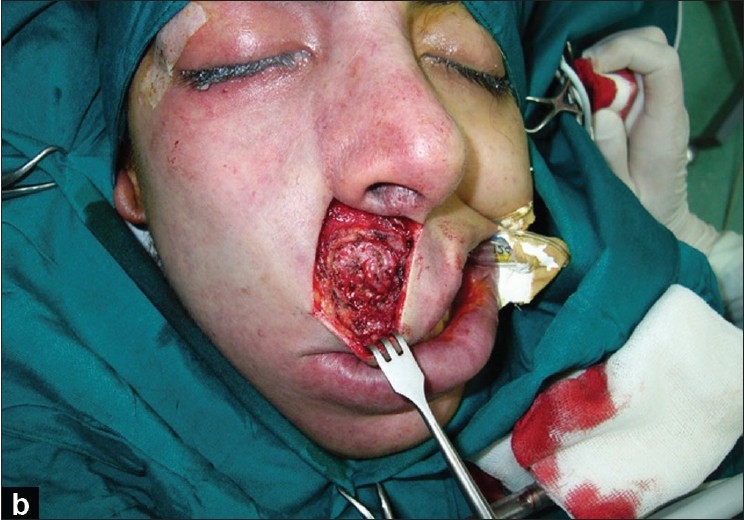
The next step in the technique is an elliptical excision of the orbicularis oris muscle and suturing with proper tension using 4-0 polyglactin 910 (Vicryl^®^) or alternately only plication of the muscle

The next step is the excision of excess skin. The amount of skin to be excised is estimated by preoperative measurements. These measurements are then adjusted during the operation. For measuring the amount of skin to be excised, the other side of the lip is used as a point of reference in order to achieve the maximum possible symmetry. In spite of this, to prevent overcorrection of the lesion, we prefer to be conservative in the amount of skin excision so some patients may require further corrective surgeries.

Wound irrigation and precise hemostasis is mandatory. Fortunately, bleeding is not a major problem during this operation. However, due to a considerable risk of bleeding, patients with high-flow vascular malformations with arterial components are not suitable candidates for this technique.

The procedure is completed by the advancement of the skin flap to adjust the vertical height of both sides of the lip and the commisures, following which the wound is closed without excessive tension in two layers using 5-0 polyglactin 910 (Vicryl^®^) and 6-0 nylon [Figure 1c]. The skin sutures are removed 1 week later.

**Figure 1c:**
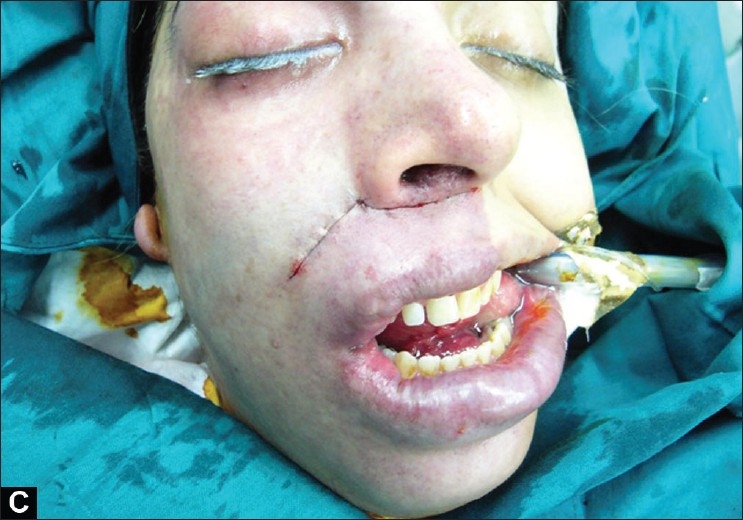
The last step in the unilateral lip lift is the closure of the wound without excessive tension in two layers using 5-0 polyglactin 910 (Vicryl^®^) and 6-0 nylon

In some cases, mucosal debulking may be necessary. In these cases, we propose a reduction cheiloplasty through the mucosal side achieved by a fusiform excision at the vermillion-mucosal line and suture using 4-0 polyglactin 910 (Vicryl^®^).

The post-operative care includes a mild compressive bandage on the lip for a period of 24 hours to limit the development of a hematoma. We also administer oral anti-inflammatory drugs (acetaminophen 300 mg four times per day) and oral antibiotics (Cephalexin 500 mg four times per day) for 7 days. The patients are advised to avoid smiling or laughing, forceful chewing, and smoking for 2 weeks.

## RESULTS

From October 2004 through October 2008, 21 patients (13 females and 8 males) underwent a unilateral upper lip lift for correction of vertical disproportion in unilateral upper lip vascular anomalies. Their mean age at first examination was 15 years, and the age range was 9 to 36 years. In 12 patients, the upper lip deformities were caused by hemangiomas. The remaining 9 patients had macrocheilia associated with vascular malformations. Five patients had isolated involvement of the upper lip. In the other patients, the cheek(s), nose, lower lip, or chin were also involved to some extent. Four patients experienced some form of functional impairment before the operation including difficulty in speaking, eating, and drinking. Eleven patients had a history of previous treatments including corticosteroids, laser, and interferon. However all of these treatments had failed to satisfy the patients.

Some patients may need revisions and further interventions as well as laser touch ups for restoration of near normal lip form. A second revisional operation was necessary in 7 of our cases. The second operation was carried out 3 months after the original unilateral upper lip lift.

We achieved nearly symmetrical vertical height of commisures in all patients with one or two procedures. Our patients experienced no problem in lip movement and function after the unilateral lip lift. In the postoperative period, only 1 patient developed a hematoma that was treated successfully with drainage and irrigation. Some transient dysesthesia of the upper lip is common in the early postoperative period but persistent dysesthesia or anesthesia was not seen.

### Case 1

The first case is an 18-year-old female who suffered from a hemangioma on the left side of her upper lip. Since she had a noticeable tumor mass and severe vertical disproportion, we planned to perform a unilateral upper lip lift and concomitant reduction cheiloplasty [Figure 2a]. Due to an incomplete correction of her problem, a second revisional operation was carried out 3 months after the unilateral upper lip lift. In a follow-up visit 6 months later, the functional and aesthetic results were satisfactory [Figure 2b].

**Figure 2a:**
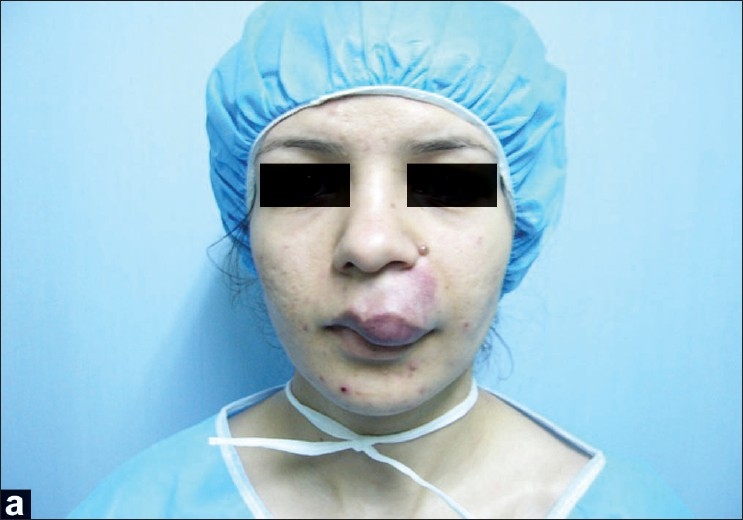
An 18-year-old female with a hemangioma on the left side of her upper lip; before the surgery

**Figure 2b:**
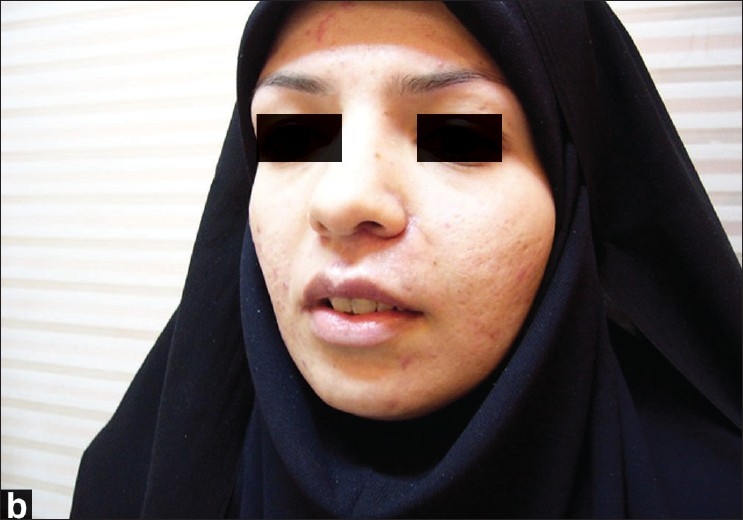
The same lady in Fig 2a after the surgery

### Case 2

A 25-year-old male with a vascular malformation on the right side of his upper lip was operated upon using the unilateral upper lip lift technique [Figure 3a]. Postoperative recovery was uneventful and the patient was discharged the day after surgery. Within 6 days following the operation, he returned to work. Six months later, the scar was found to be aesthetically acceptable and the unilateral long lip had been corrected [Figure 3b].

**Figure 3a:**
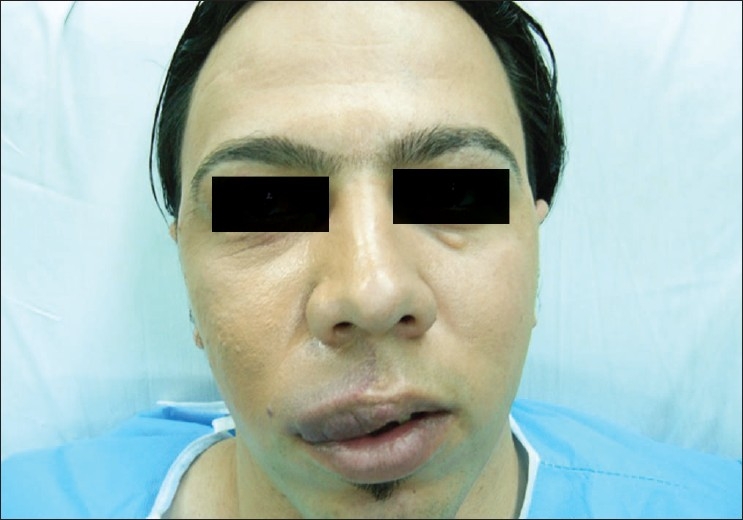
A 25-year-old male with a vascular malformation on the right side of his upper lip; before the surgery

**Figure 3b:**
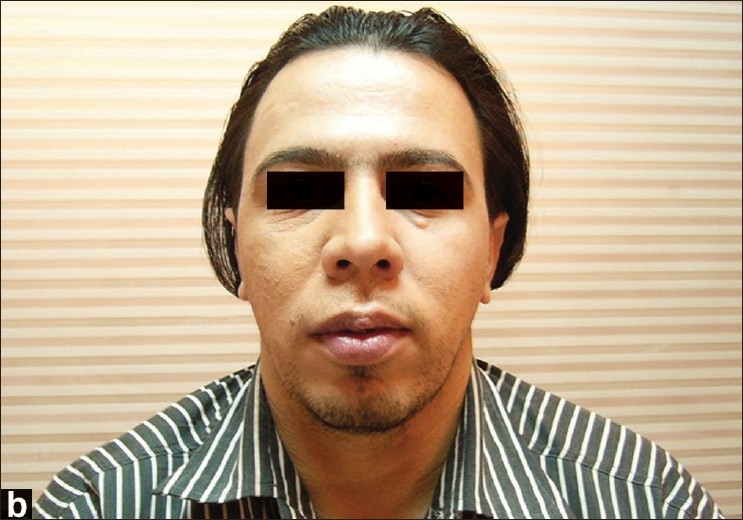
A 25-year-old male with a vascular malformation on the right side of his upper lip; after the surgery

### Case 3

A 22-year-old female with a vascular malformation on the right side of her upper lip was considered for surgical treatment. We planned a unilateral upper lip lift and concomitant reduction cheiloplasty and rhinoplasty [Figure 4a]. Postoperative recovery was uneventful and the patient was discharged the day after the surgery. Six months later, the scar was aesthetically acceptable and the unilateral long lip was corrected [Figure 4b].

**Figure 4a:**
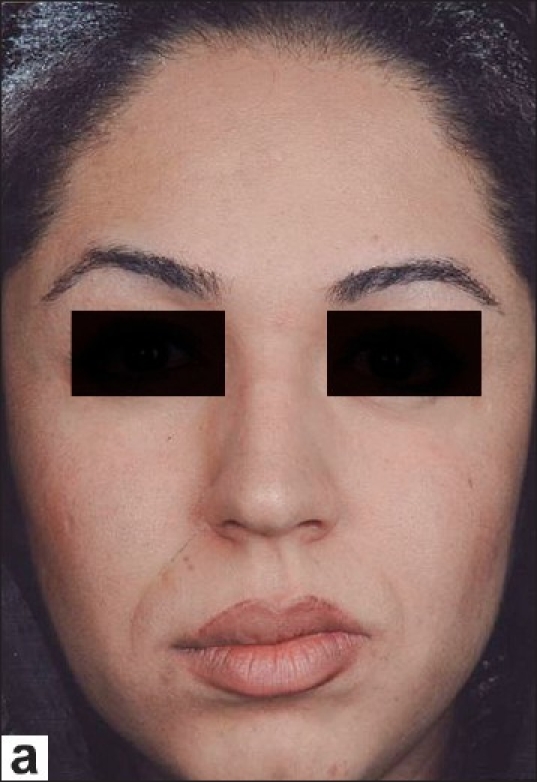
A 22-year-old female with a vascular malformation on the right side of her upper lip; before the surgery

**Figure 4b:**
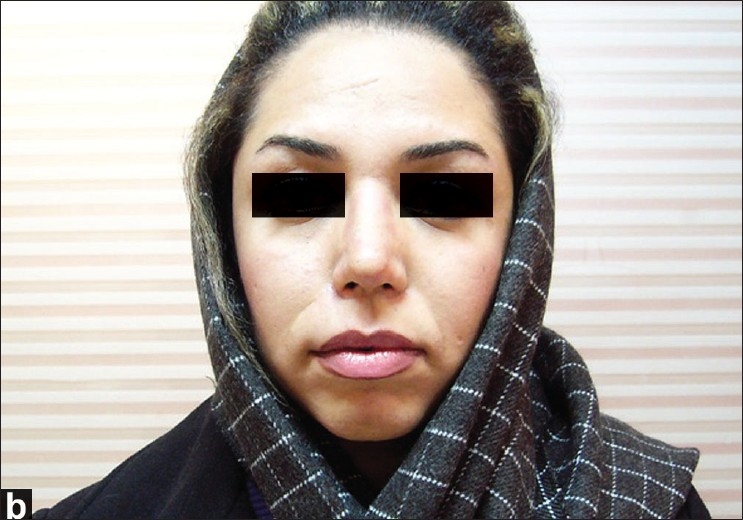
A 22-year-old female with a vascular malformation on the right side of her upper lip; after the surgery

## DISCUSSION

Congenital vascular anomalies including vascular malformations and hemangiomas that fail to regress pose a difficult challenge both for the surgeon and the patient. Through the years, different surgical techniques have been proposed for the lesions that primarily involve the lips. These techniques include generally sequential debulkings through skin or mucosal incisions.[6,7,13,14] Our review of the Medline database revealed that there are no reported accounts of performing a procedure like the unilateral upper lip lift for the treatment of upper lip vascular anomalies. As we mentioned before, due to the high risk of bleeding, our technique is not appropriate for high-flow vascular malformations with an arterial component.

The upper lip lift is a cosmetic procedure that elevates the position of the upper lip with respect to the teeth giving a broader smile. The result of the lip lift is a more youthful and pleasant shape to the mouth and lips. A central lip lift is achieved either through a subnasal approach (the bull's horn approach) or through an incision above the vermilion (supravermilion or Gillies approach).[14,15]

In the classic upper lip lift, the incision is limited to the subnasal area. We have adopted a variation of the subnasal approach with the primary excision starting from the subnasal area in midline and extending into the perialar and nasolabial area (unilateral upper lip lift). An elliptical excision of the orbicularis oris muscle or alternately plication of the muscle is another step unique to our technique.

Precise skin excision based on preoperative measurements and intraoperative assessment is critical. Inadequate or excessive skin excision leads to asymmetry in vertical heights or mouth angles. Our experience has shown that the advantages of our approach are a less obvious surgical scar, wider exposure for better debulking, and the ability to achieve commissure lift. Since the timing of involution of hemangiomas cannot be predicted, the judgment in choosing the optimal time for surgical intervention is a difficult one.
